# The clastogenicity of 4NQO is cell-type dependent and linked to cytotoxicity, length of exposure and p53 proficiency

**DOI:** 10.1093/mutage/gev069

**Published:** 2015-09-11

**Authors:** Katja Brüsehafer, Bella B. Manshian, Ann T. Doherty, Zoulikha M. Zaïr, George E. Johnson, Shareen H. Doak, Gareth J. S. Jenkins

**Affiliations:** In Vitro Toxicology Group, Institute of Life Science, College of Medicine, Swansea University, Singleton Park, Swansea SA2 8PP, UK and; ^1^Drug Safety and Metabolism, AstraZeneca, Unit 310, Darwin Building, Cambridge Science Park, Milton Road, Cambridge CB40WG, UK

## Abstract

4-Nitroquinoline 1-oxide (4NQO) is used as a positive control in various genotoxicity assays because of its known mutagenic and carcinogenic properties. The chemical is converted into 4-hydroxyaminoquinoline 1-oxide and gives rise to three main DNA adducts, *N*-(deoxyguanosin-8-yl)-4AQO, 3-(desoxyguanosin-*N* ^2^-yl)-4AQO and 3-(deoxyadenosin-*N* ^6^-yl)-4AQO. This study was designed to assess the shape of the dose–response curve at low concentrations of 4NQO in three human lymphoblastoid cell lines, MCL-5, AHH-1 and TK6 as well as the mouse lymphoma L5178Y cell line *in vitro*. Chromosomal damage was investigated using the *in vitro* micronucleus assay, while further gene mutation and DNA damage studies were carried out using the hypoxanthine–guanine phosphoribosyltransferase forward mutation and comet assays. 4NQO showed little to no significant increases in micronucleus induction in the human lymphoblastoid cell lines, even up to 55±5% toxicity. A dose–response relationship could only be observed in the mouse lymphoma cell line L5178Y after 4NQO treatment, even at concentrations with no reduction in cell viability. Further significant increases in gene mutation and DNA damage induction were observed. Hence, 4NQO is a more effective point mutagen than clastogen, and its suitability as a positive control for genotoxicity testing has to be evaluated for every individual assay.

## Introduction

Genetic toxicology involves the assessment of a substance’s ability to induce DNA damage, which is an essential consideration for human health risk assessment because DNA damage is an underlying cause of mutations that have the potential to initiate carcinogenesis. It is essential to investigate and understand the biological significance of genotoxic effects of chemicals at the low-dose exposure range to improve human health risk assessment and to establish if DNA reactive compounds follow linear or non-linear dose–response relationships.

Traditionally, high concentrations of genotoxins were used in *in vitro* testing to ensure that DNA damaging effects were identified and because of the assumption that genotoxins follow a linear relationship that was extrapolated back to the low-dose region (1). However, in recent years, the linear model was challenged (1–3), and it became apparent that inappropriately high concentration in genetic toxicology testing was responsible for many of the false-positive results in Stage 1 *in vitro* testing (1,4). *In vitro* genetic toxicity assays have been extensively used in safety assessment studies and have contributed to our understanding of the dose–response relationships of aneugens, clastogens and point mutagens (5). Genotoxicants can interact with DNA by various mechanisms, such as direct interaction of the compound with DNA, interaction of the compound with cellular components that cause indirect DNA damage and DNA damage can also be induced through activation of the compound by cellular metabolism to produce products, which are capable to subsequently interact with DNA (6).

4-Nitroquinoline 1-oxide (4NQO) is a known mutagen and carcinogen and is therefore used in various genotoxicity assays as a positive control (7). The chemical was first synthesised in 1942, and its carcinogenicity was first demonstrated in 1957 (8,9). Since then, 4NQO has widely been used in experimental oncology as a potent carcinogen (10). It is known that 4NQO induces cancer in various tissues in mice and rat, examples of which are lung, pancreas and stomach (11). Chemically, 4NQO is comprised of two polar groups, the *N*-oxide and nitro group. It is therefore susceptible to nucleophilic attack in chemical reactions (9). The enzymatic reduction of its nitro group is believed to cause 4NQO’s carcinogenic effect. 4NQO is converted into 4-hydroxyaminoquinoline 1-oxide (4HAQO) and 4-aminoquinoline 1-oxide (4AQO); however, only 4HAQO is believed to be carcinogenic (11). 4NQO and its reduced metabolite 4HAQO are able to bind to cellular macromolecules, such as nucleic acid and protein (12). The activated 4HAQO gives rise to three main adducts by reacting with purines, but not with pyrimidines (12); of these adducts, two are located within the guanine base and another adenine adduct is also formed. The structures of adducts identified were nucleic acid bases, nucleosides or nucleotides of *N*-(deoxyguanosin-8-yl)-4AQO, 3-(desoxyguanosin-*N* ^2^-yl)-4AQO and 3-(deoxyadenosin-*N* ^6^-yl)-4AQO (11,13). The dGuo-C8-AQO adduct results from an attack of the nitrenium ion on the C8 position of guanine, whereas the dGuo-N2-AQO and the dAdo-N6-AQO adducts results from a carbocation attack on the guanine N2 and the adenine N6 positions, respectively (11). It is known that 4NQO reacts primarily with DNA at the N2 and C8 position of guanine and to a smaller extent at the N6 position of adenine (14). Further, 8-hydroxydeoxyguanosine (8-OH-dG) formation has been reported in cellular DNA treated with 4NQO (13,15), and it has been shown that 4NQO also induces oxidative stress and generates reactive oxygen species (ROS), such as superoxide radicals or hydrogen peroxide (16,17).

This study was designed to assess the shape of the dose–response curve at low concentrations of 4NQO in human and mouse cells *in vitro* and to investigate the effects of using different study designs on the points of departure (PoD) and genotoxic potency. Chromosomal damage was investigated using the *in vitro* micronucleus (MN) assay, while further gene mutation and DNA damage studies were carried out using the hypoxanthine–guanine phosphoribosyltransferase (HPRT) forward mutation and comet assays. Comparative studies were performed in two laboratories, Swansea University, Swansea, UK and AstraZeneca, UK.

## Materials and methods

### Test agent

4NQO was acquired from Sigma–Aldrich (Dorset, UK) and dissolved in dimethyl sulfoxide (DMSO; Fisher Scientific, Loughborough, UK). Before use, the chemical was freshly diluted from a stock solution (2.5mg/ml aliquots at Swansea University and 0.019mg/ml aliquots at AstraZeneca) with DMSO.

### Cell lines and culture conditions

At Swansea University, the human lymphoblastoid cell lines, MCL-5, AHH-1 and TK6, were utilised. AHH-1 is a human lymphoblastoid TK^+/−^ cell line that constitutively expresses a high level of native CYP1A1 (18). AHH-1 cells carry a heterozygous mutation in the TP53 locus (19–21). The human lymphoblastoid cell line MCL-5 is derived from AHH-1 by stable transfection with human cytochromes (CYP1A2, CYP2A6, CYP3A4 and CYP2E1) and microsomal epoxide hydrolase (18). These cytochromes and a hygromycin B resistance gene are carried as cDNAs in plasmids incorporated into the cell. Further, MCL-5 cells carry, like AHH-1 cells, a heterozygous mutation in the TP53 locus. The human lymphoblastoid cell line TK6 is a derivative of the WIL-2 cell line and contains the wild-type TP53 gene. For the experiments carried out at AstraZeneca, TK6 and the mouse lymphoma cell line L5178Y were used. L5178Y cells are known for their dysfunctional p53 activity (22,23). TK6 cells showed a composite karyotype of 47 XY, +der3t(3,21), +der13t(13,22) and −14+der14t(14,20), while L5178Y cells showed a composite karyotype of 40 X0 der5t(5,15), der9t(9,6) Robertsonian fusion 12 and 13, del 14+15(t15,5), +15(t15,18,14), der15, −15q and der 18t(18,6).

The average doubling time of TK6 cells in both laboratories was ~16 to 18h. The doubling time of AHH-1 and MCL-5 cells was ~22 to 24h, whereas the L5178Y doubling time was ~11.5h.

At Swansea University, all cell lines were cultured in RPMI 1640 (GibCo® Invitrogen, Paisley, UK) supplemented with 10% donor horse serum (BD Gentest, Oxford, UK) and 1% l-glutamine (GibCo® Invitrogen, Paisley, UK). Cells were maintained at 37°C in a humidified atmosphere of 5% CO_2_ in air. For MCL-5 cells, hygromycin B (Merck, Darmstadt, Germany) in acetic acid (35mM) was added at each passage to a final concentration of 200 µg/ml to ensure plasmid retention (24).

TK6 cells at AstraZeneca were cultured in RPMI 1640 (Sigma–Aldrich, Dorset, UK) supplemented with 10% heat-inactivated donor horse serum (Gibco® Invitrogen, Paisley, UK), 2mM l-glutamine (Gibco® Invitrogen, Paisley, UK), 2mM sodium pyruvate (Gibco® Invitrogen, Paisley, UK), 200 IU/ml penicillin (Gibco® Invitrogen, Paisley, UK) and 200 µg/ml streptomycin (Gibco® Invitrogen, Paisley, UK) at 37°C, 5%CO_2_. L5178Y cells were cultured in RPMI 1640 supplemented with 10% heat-inactivated donor horse serum, 2mM l-glutamine, 2mM sodium pyruvate, 1% Pluronic F68 (Gibco® Invitrogen, Paisley, UK), 200 IU/ml penicillin and 200 µg/ml streptomycin at 37°C, 5%CO_2_.

### Treatment with test agent

For the *in vitro* cytokinesis-block micronucleus (CBMN) assay, AHH-1 and MCL-5 cells were treated with 4NQO over a range of concentrations up to 0.7 µg/ml for 4h, followed by one cell cycle of cytochalasin B (4.5 µg/ml for MCL-5 cells; 6 µg/ml for AHH-1 cells). TK6 cells were treated with 4NQO over a range of concentrations up to 0.03 µg/ml for 4, 24 or 48h, respectively, followed by one cell cycle of cytochalasin B (4.5 µg/ml). Further, TK6 cells were treated for 24h with 4NQO for the HPRT assay study with 0.0009, 0.003, 0.01 and 0.02 µg/ml 4NQO. Medium was used as negative control, whereas DMSO was used as the solvent control. The experiments were carried out in triplicate.

For the mononucleated *in vitro* MN assay, L5178Y cells were treated with 4NQO for 4h, followed by 24-h recovery time over a range of concentrations up to 0.05 µg/ml. Further, TK6 cells were treated with 4NQO for 4h plus 40-h recovery period and 24h plus 24-h recovery time over a range of concentrations up to 0.03 µg/ml. For the comet assay, TK6 cells were treated with 4NQO for 3h over a range of concentrations up to 0.06 µg/ml 4NQO because of previous experiments conducted at AstraZeneca (data not shown). The experiments were carried out in duplicate.

The treatment regimes chosen covered a range from producing 55±5% cytotoxicity to little or no toxicity, as higher levels may induce chromosome damage as a secondary effect of cytotoxicity (7).

### The *in vitro* MN assay

The CBMN assay was carried out at Swansea University. Ten-millilitre cell suspensions were seeded at 1×10^5^ cells/ml for 24h at 37°C, 5%CO_2_ prior to treatment. Details of the assay are described by Brüsehafer *et al.* (25).

The *in vitro* MN test (mononucleated assay) procedure routinely performed at AstraZeneca used the semi-automated MN scoring system Metafer (MetaSystems, Germany). Ten-millilitre cell suspensions were seeded at 2×10^5^ cells/ml and immediately treated with 1% v/v solvent control and the test chemical at 37°C, 5%CO_2_. After the different incubation times, all of the cell suspensions were removed from each flask and transferred into appropriately labelled tubes. The cells were centrifuged (5min, 200 × *g*), the supernatant was discarded and the pellets washed with phosphate-buffered saline (PBS), before re-suspending the cells in 10-ml fresh media. The cells were cytospun onto polished glass slides. Shandon mega-funnels (8min, 1000rpm) were used to harvest cells for the Metafer System. The cells were fixed in 100% methanol for 15min. For the semi-automated scoring protocol, the cells were stained for 10min with a 4′,6-diamidino-2-phenylindole + vectashield (Vector Lab., Inc., UK) solution and scanned with the Metafer4 software. All experiments carried out at AstraZeneca were run in duplicate. One thousand mononucleated cells were scored per replicate.

### Cytotoxicity and cytostasis

Relative population doubling (RPD) was used to measure cytotoxicity and cytostasis in the absence of cytochalasin B. Therefore, satellite cultures were seeded as described previously for the CBMN assay (25). Cells were counted with the Beckman coulter counter (Z1 Coulter® Particle Counter, Beckman, UK) 1h before treatment (control cultures) and at the cell harvest stage (treated cultures).

RPD was calculated as follows:

$$\text{RPD}=\frac{\text{Number of population doublings in treated cultures}}{\text{Number of population doublings in control cultures}}\times 100$$

where

$$\text{Population doubling}=\frac{\text{[log }(\text{post-treatment cell number}/\text{initial cell number})]}{\text{Log 2}}$$

### The HPRT gene mutation assay

The first step of the HPRT assay was the mutant cleansing stage to remove background mutants within the TK6 cell population. Therefore, hypoxanthine–aminopterin–thymidine (HAT) was added to the TK6 culture (4–5×10^5^ cells/ml), and the cells were grown for 3 days. After 3 days, the HAT medium was washed off with PBS. Afterwards, the cells were re-suspended in 50-ml fresh medium, and hypoxanthine–thymidine (HT) was added to the culture medium for 24h. After washing off the HT medium with PBS, TK6 cell suspensions were seeded at 1×10^5^ cells/ml for 24h at 37°C, 5%CO_2_. The cell suspensions were then treated with 4NQO, and after the treatment period, 4NQO was then washed off with PBS, new medium was added and the cells were sub-cultured to 1.2–1.5×10^5^ cells/ml in 50-ml flasks (Day 1). The cells were sub-cultured and left to grow for 13 days in total to enable expression of the HPRT^−^ mutants. Cells were sub-cultured at Days 1, 3, 5, 7, 9 and 11. After the phenotypic expression period at day 13, the cells were added to 96-well micro plates.

For mutant frequency, TK6 cells with 2.4×10^7^ cells in 60ml were treated with 240 µl of 6-thioguanine (0.6 µg/ml) (6-TK) for selection. Five plates per dose were used with 100 µl of cell suspension in each well. Plates were scored for colony formation after 14 days of continuous incubation at 37°C, 5%CO_2_. Scoring criteria specified that only colonies with 20+ cells in diameter were scored. Further, it was ensured that separate colonies were clearly apart, taking clonal expansion into account. These experiments were carried using three separate stock solutions, but with the same cell passage.

### The *in vitro* comet assay +/−hOGG1

For the *in vitro* comet assay, TK6 cells were treated with 4NQO that was then washed off with PBS and the cells were re-suspended in fresh media. The cell number was adjusted to 1.5×10^5^ cells/ml. After that, 1ml of the cell suspensions were sampled into labelled Eppendorf tubes and washed with 1ml cold 1× PBS. After centrifugation, the supernatant was removed from the pellet and the cells were re-suspended with 200-µl low-melting agarose and 3×40 µl drops, resulting in three gels, were added per glass slide (pre-coated with 0.5% agarose) and covered with a coverslip. Slides were then placed on a cold tray, which allowed for gels to set after which the coverslips were removed and the slides were placed in cold lysis solution [2.5M NaCl, 100mM disodium ethylenediaminetetraacetate dihydrate (Na_2_EDTA), 10mM Tris buffer (pH 10), 10% DMSO, 1% Triton X-100] in a dark container over night at 4°C. Following lysis, the slides were washed twice for 5min in 1× buffer F [40mM 4-(2-hydroxyethyl)-1-piperazineethanesulfonic acid, 0.1M KCl, 0.5mM ethylenediaminetetraacetic acid and 0.2mg/ml bovine serum albumin (pH 8.0)] at room temperature. hOGG1 (+hOGG1) or buffer F (−hOGG1) (60 µl) were then added to each slide, topped up with a coverslip and incubated in a humidified box at 37°C for 10min. After removing the coverslips, the slides were placed in an electrophoresis platform and covered with electrophoresis buffer [1mM Na_2_EDTA, 0.3M NaOH (pH 13)] for 20min at 4°C to allow the DNA to unwind. Afterwards, the electrophoresis was performed at 0.7V/cm, 300 mA for further 20min at 4°C. To neutralise, the slides were removed from the electrophoresis platform and immersed in three changes of neutralisation buffer [0.4M Tris–HCl (pH 7.5)] for 5min each time at room temperature. Finally, the slides were stained with 60-µl propidium iodide (20 µg/ml) for 30min, before scoring using a Comet IV capture system (Perceptive Instruments Ltd., Haverhill, UK). Fifty nuclei per gel were scored and the tail intensity that is defined as the percentage of DNA migrated from the head of the comet into the tail was measured for each nucleus scored (26). The experiment was run in duplicate.

### Data analysis

Error bars are represented as standard deviation of the mean. Student’s paired *t*-test (*n* = 2) or a one-way analysis of variance approach (*n* = 3), followed by a two-sided Dunnett’s *post hoc* test with log-transformed data based on Kolmogorov–Smirnov test for normality and Bartlett’s test for homogeneity of variance (27), were used to determine if any of the treatment concentrations were significantly different from the control. BM dose (BMD) approach with covariate analysis was carried out to assess the MN data. BMD values were derived using the dose–response modelling software package PROAST version 50.8, using covariate analysis to improve precision and provide information on potency (28,29; G. E. Johnson *et al*., in preparation).

## Results

4NQO is used as a positive control in various *in vitro* genotoxicity assays because of its known mutagenic and carcinogenic properties. The aim of this study was to investigate the low dose–response relationships of 4NQO in human lymphoblastoid cells to calculate and compare PoDs for genotoxic activity. The chosen exposure and recovery times were optimised using empirical experiments bearing in mind the need to keep cytotoxicity levels below 55±5% as dying cells can confound the MN assay results.

### Chromosome damage induction in p53 compromised MCL-5 and AHH-1 cells and the mouse lymphoma cell line L5178Y

**Figure 1.**
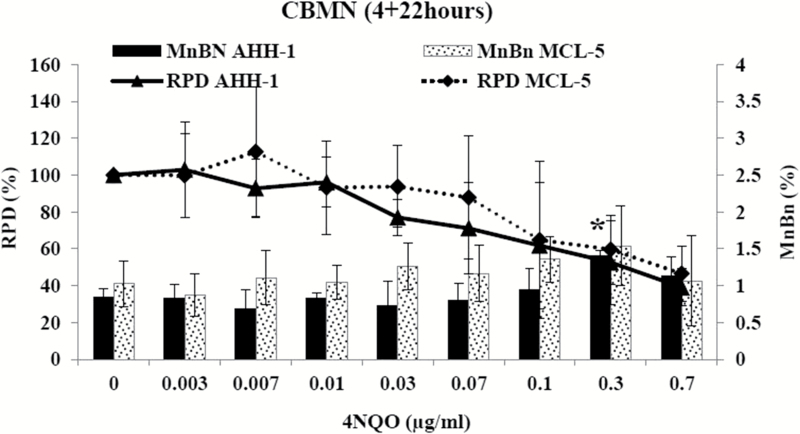
Effect of 4NQO on AHH-1 and MCL-5 cells using the *in vitro* CBMN assay. AHH-1 and MCL-5 cells were treated with 4NQO for 4h, followed by 22h of cytochalasin B. Columns: percentage micronucleated binucleated cells, lines: RPD. Values represent mean ± standard deviation (*n* = 3). *P < 0.05 (Dunnett’s *post hoc* test).

**Figure 2.**
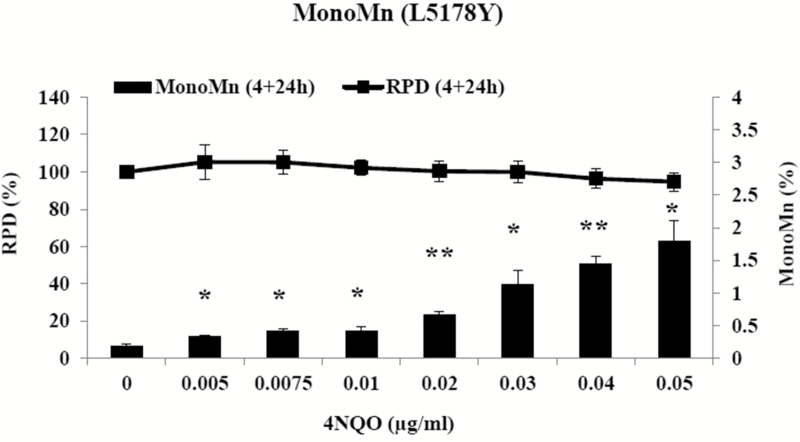
Effect of 4NQO on L5178Y cells using the *in vitro* MN assay. L5178Y cells were treated with 4NQO for 4h, followed by a 24-h recovery period. Column: percentage micronucleated mononucleated cells, line: RPD. Values represent mean and range of duplicate experiments. *P < 0.05, **P < 0.01 (Student’s paired *t*-test).

To evaluate if 4NQO requires metabolic activation and to investigate the role of p53 proficiency, the metabolically competent human cell line MCL-5 and the parent cell line AHH-1, which holds some degree of metabolic competence, were treated with 4NQO at Swansea University. Both these cell lines have a heterozygous p53 mutation in codon 282. At AstraZeneca, the mouse lymphoma cell line L5178Y (with dysfunctional p53 function) was treated with 4NQO. L5178Y cells are frequently used for genetic toxicity studies. Short-term treatments with and without prolonged recovery times were used for the investigation. AHH-1 cells treated with 4NQO for 4h over a range of concentration up to 0.7 µg/ml, followed by one cell cycle of cytochalasin B (22h) showed significant increases in MN induction only at 0.3 µg/ml. However, MCL-5 cells showed no significant increases in MN induction for the short-term treatment over the range of concentrations chosen. At 0.7 µg/ml, high toxicity levels were observed in AHH-1 and MCL-5 (61% in AHH-1 and 53.5% in MCL-5) cells (Figure 1). L5178Y cells were treated with 4NQO for 4h followed by a prolonged recovery time of 24h (two cell cycles) prior to harvesting, over a concentration range up to 0.05 µg/ml. The average doubling time of L5178Y cell was 11.5h, hence they grow much faster than the other cell lines used. The cells showed significant increases in MN induction at the lowest concentration selected (0.005 µg/ml) and above. No reduction in cell viability could be observed over the range of concentrations tested (Figure 2). The experiments showed that 4NQO required no metabolic activation to induce chromosomal damage.

**Figure 3.**
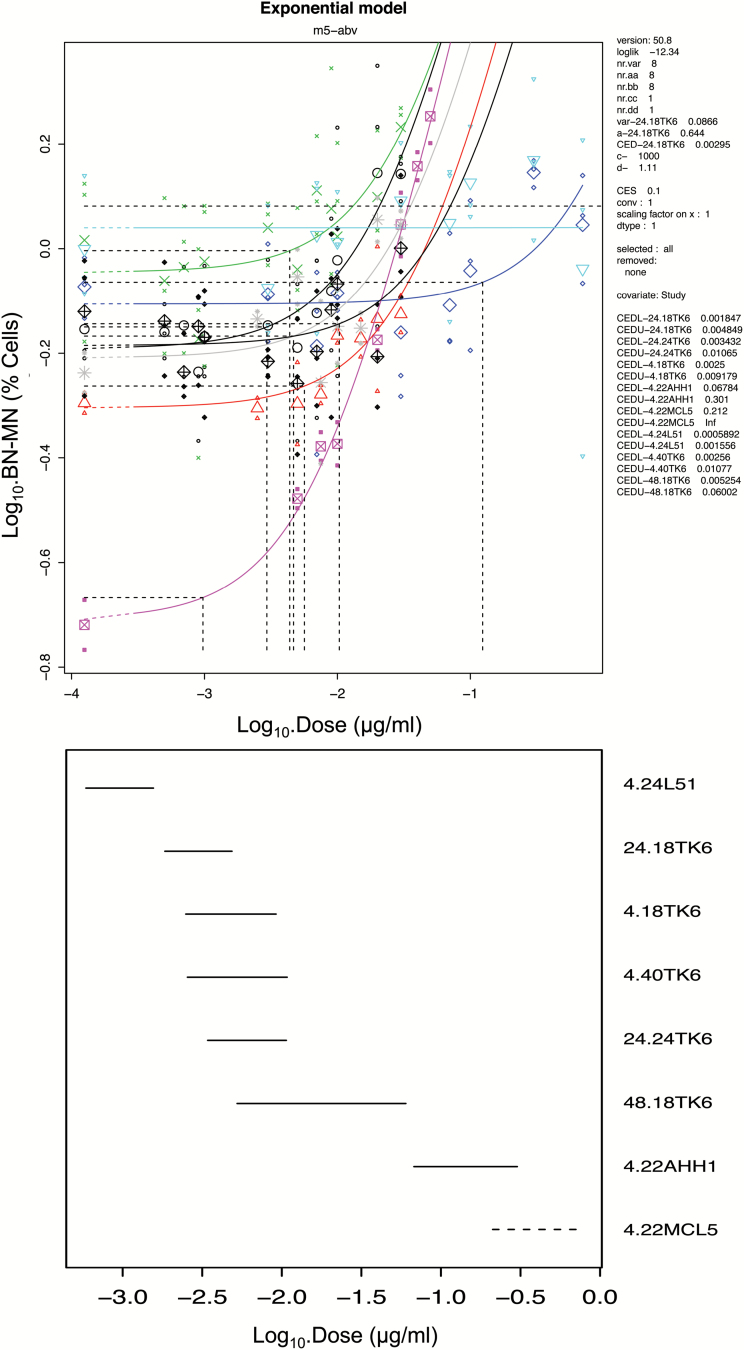
BMD covariate analysis of MN dose–response data for 4NQO in human lymphoblastoid cell lines AHH-1, MCL-5 and TK6 and the mouse lymphoma cell line L5178Y. The upper panel shows the dose–response relationships for each time point/cell type analysed (e.g. ‘24.18TK6’ represents 24-h 4NQO exposure with 18-h recovery in TK6 cells) with the critical effect size (CES = benchmark response [BMR]), and values used for the model parameters var, a and c during PROAST analysis displayed to the right alongside the critical effect doses (CED = BMD) and upper and lower 90% confidence intervals (CEDU/CEDL) determined for each dose response. The lower panel plots the BMDL_10_–BMDU_10_ 90% confidence interval width for each dose response. PROAST version 50.8 was used with a BMR of 10% and the exponential model ‘m5-abv’ was selected as an appropriate model for this data set. Data/covariate level model fits are shown in supplementary Figure 1, available at *Mutagenesis* Online.

The dose responses were further assessed by using BMD modelling with covariate analysis in PROAST version 50.8 (Figure 3; supplementary Figure 1, available at *Mutagenesis* Online). BMD modelling utilises a set of statistical models to estimate a defined increase above its control response values (benchmark response), along with its confidence limits. For the AHH-1 data after treatment with 4NQO, a BMD_10_ (10% increase above the background) was calculated at 0.1238 μg/ml with confidence intervals between 0.0678 and 0.3007 μg/ml, respectively. No dose response was observed for the MCL-5 data; however, the covariate approach enabled the derivation of BMD_10_ and BMDL_10_ metrics at 46.4590 and 0.2120, respectively, along with an infinite BMDU_10_. The L5178Y data set revealed a BMD_10_ at 0.0010 μg/ml with a BMDL_10_ and BMDU_10_ of 0.0006 and 0.0016 μg/ml, respectively (Table 1).

**Table 1.** BMD values for the MN end point induced by 4NQO derived from Figure 3

	4+24h L5178Y	24+18h TK6	4+18h TK6	4+40h TK6	24+24h TK6	48+18h TK6	4+22h AHH-1	4+22h MCL-5
BMD_10_	0.0010	0.0029	0.0044	0.0047	0.0057	0.0103	0.1238	46.4590
BMDL_10_	0.0006	0.0019	0.0025	0.0026	0.0034	0.0053	0.0678	0.2120
BMDU_10_	0.0016	0.0049	0.0092	0.0108	0.0107	0.0597	0.3007	Inf

### Chromosome damage induction in p53 competent TK6 cells

**Figure 4.**
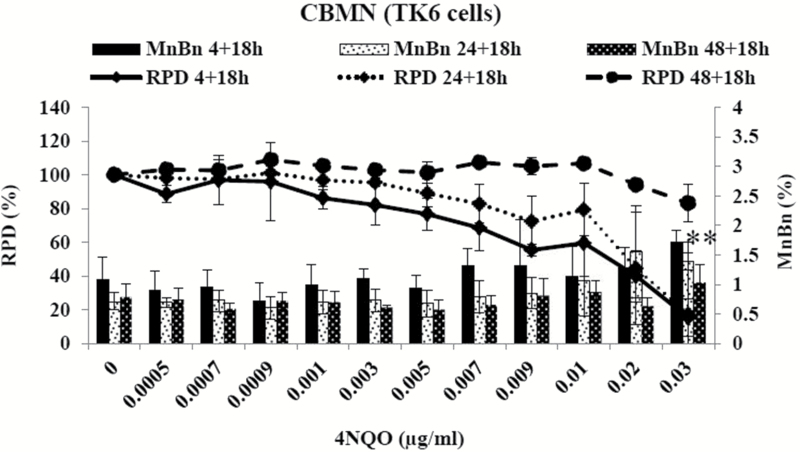
Effect of 4NQO on TK6 cells using the *in vitro* CBMN assay. TK6 cells were treated with 4NQO for 4, 24 or 48h, followed by 18h of cytochalasin B. Columns: percentage micronucleated binucleated cells, lines: RPD. Values represent mean ± standard deviation (*n* = 3). **P < 0.01 (Dunnett’s *post hoc* test).

The *in vitro* MN assay was performed in TK6 cells following Organisation of Economic Cooperation and Development guidelines utilising short and extended treatment periods (3). Exposure of TK6 cells with 4NQO for 4h followed by one cell cycle of cytochalasin B (18h) showed no significant increases in MN induction over the treatment range chosen (up to 0.03 µg/ml). However, high levels of cell death and cytostasis (60.1–84.7%) was observed at 0.02 µg/ml of 4NQO and above (Figure 4). Extended treatments of 24- and 48-h exposure to 4NQO followed by one cell cycle of cytochalasin B (18h) were subsequently performed. TK6 cells treated with 4NQO for 24h showed significant increases in MN frequencies only at 0.03 µg/ml. However, high toxicity levels were again observed at 0.02 µg/ml (55.5%) of 4NQO (Figure 4). No significant increases in MN induction were observed when TK6 cells were exposed for 48h (+18h) to 4NQO; however, at 0.03 µg/ml, ~30% cell death and cytostasis were observed (Figure 4).

**Figure 5.**
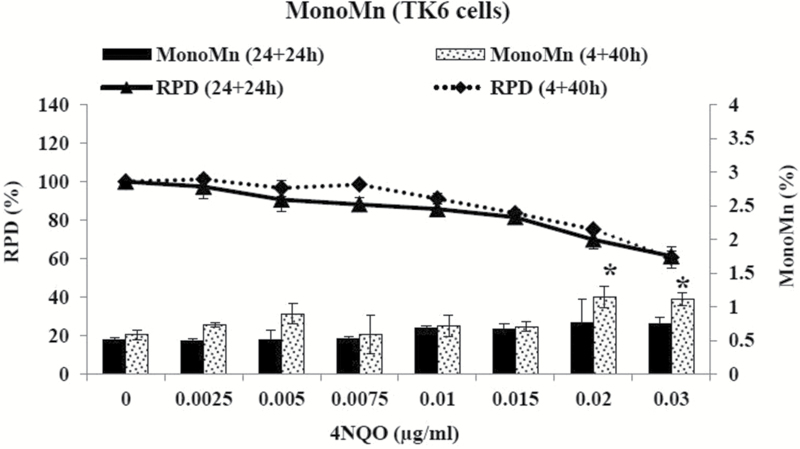
Effect of 4NQO on TK6 cells using the *in vitro* MN assay. TK6 cells were treated with 4NQO for 24h, followed by a 24-h recovery period, or for 4h, followed by a 40-h recovery period. Columns: percentage micronucleated mononucleated cells, lines: RPD. Values represent mean and range of duplicate experiments. *P < 0.05 (Student’s paired *t*-test).

In a previous study (30), it was shown that an extended recovery time might play a critical role in MN induction, at least in compounds with direct DNA reactivity, such as mitomycin C. Therefore, TK6 cells were further treated with 4NQO for 24h, followed by a 24-h (one and a half cell cycles) recovery period before harvesting. No significant increases in MN induction were observed however over a range of concentrations between 0 and 0.03 µg/ml 4NQO. About 40% (38.8%) cell death and cytostasis were observed at the top concentration (0.03 µg/ml) (Figure 5). Further, a short-term treatment of 4h with an extended recovery period of over two cell cycles (40h) was also performed. Significant increases in MN induction were observed at 0.02 and 0.03 µg/ml 4NQO. At the highest dose, 39.5% toxicity was reached (Figure 5). Hence, recovery period appeared to be important for 4NQO-induced MN induction.

Subsequently, the dose responses were further assessed by BMD modelling (Figure 3; supplementary Figure 1, available at *Mutagenesis* Online). For the TK6 data after treatment with 4NQO for 4h followed by 18h of cytochalasin B, a BMD_10_ was calculated at 0.0044 μg/ml with BMDL_10_ at 0.0025 μg/ml and BMDU_10_ at 0.0092 μg/ml. TK6 cells treated with 4NQO for 24 and 18h of cytochalasin B revealed a BMD_10_ at 0.0029 μg/ml with BMDL_10_ at 0.0019 and BMDU_10_ at 0.0049 μg/ml, respectively, whereas TK6 cells treated with 4NQO for 48 and 18h of cytochalasin B revealed a BMD_10_ at 0.0103 μg/ml, BMDL_10_ at 0.0053 μg/ml and BMDU_10_ at 0.0597 μg/ml. For the TK6 cells with an extended recovery time of 24h after 24-h 4NQO treatment, a BMD_10_ was calculated at 0.0057 μg/ml with a BMDL_10_ and BMDU_10_ at 0.0034 and 0.0107 μg/ml, respectively. TK6 cells with an extended recovery time of 40h after 4-h 4NQO treatment revealed a BMD_10_ at 0.0047 μg/ml with a BMDL_10_ and BMDU_10_ at 0.0026 and 0.0108 μg/ml, respectively (Table 1). The BMD covariate approach ranked and grouped 4NQO by potency in the different culture conditions. It was shown that 4NQO in TK6 cells were very closely grouped (Figure 3, supplementary Figure 1, available at *Mutagenesis* Online).

To summarise, 4NQO showed little to no significant increases in MN induction in the human lymphoblastoid cell lines TK6, AHH-1 and MCL-5, even up to 55±5% toxicity. However, a dose–response relationship was observed in the mouse lymphoma cell line L5178Y after 4NQO treatment, even at concentrations with no reduction in cell viability, indicating an important role of the tumour suppressor gene TP53.

### HPRT gene mutation and DNA damage induction in TK6 cells

Due to the widespread use of 4NQO as a positive control in genotoxicity testing and the lack of convincing positive results in many of the above MN studies, we employed the HPRT and comet assays to investigate the induction of gene mutations and DNA damage by 4NQO.

**Figure 6.**
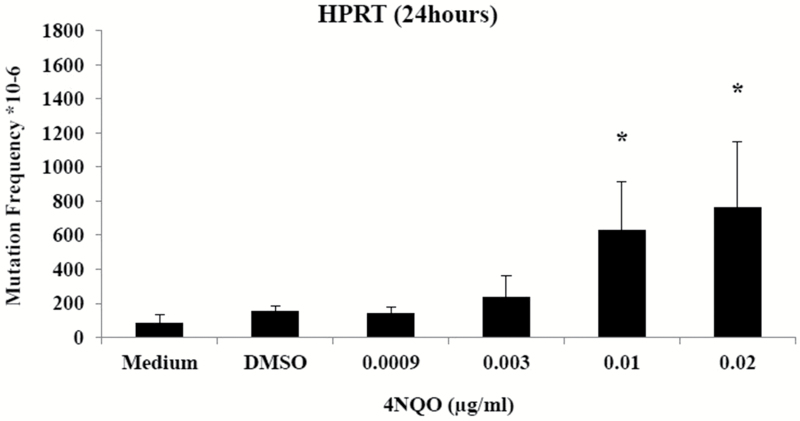
HPRT gene mutation frequency (number of 6TG resistant clones/10^6^ clone-forming cells) in TK6 cells treated with 24h of 4NQO. Values represent mean ± standard deviation (*n* = 3). *P < 0.05 (Dunnett’s *post hoc* test).

For the HPRT assay, TK6 cells were treated with 4NQO for 24h up to 0.02 µg/ml. Significant increases in gene mutation were observed at 0.01 and 0.02 µg/ml 4NQO (Figure 6). These are lower concentrations than any of the MN-inducing doses in the above experiments (0.02 µg/ml 4 + 40h). Hence, 4NQO might be a much more effective point mutagen than clastogen.

**Figure 7.**
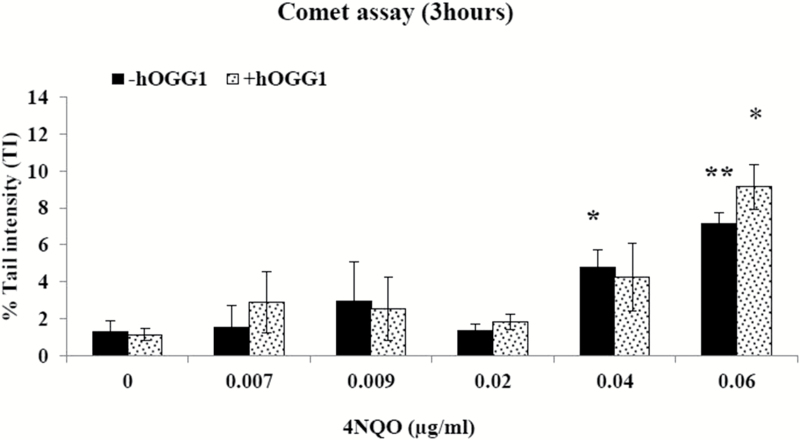
The *in vitro* comet assay. The effect of hOGG1 on DNA tail intensity, following treatment of TK6 cells with 3h of 4NQO. Values represent mean and range of duplicate experiments. *P < 0.05, **P < 0.01 (Student’s paired *t*-test).

With the comet assay, low levels of DNA damage can be detected. The assay was performed with and without hOGG1. hOGG1 is an endonuclease that recognises oxidative DNA damage (26). TK6 cells were treated with 4NQO for 3h showed significant increases in DNA damage at 0.04 and 0.06 µg/ml 4NQO, without hOGG1 and at 0.06 µg/ml 4NQO with hOGG1 (Figure 7). In summary, 4NQO induced gene mutation and DNA damage at the higher range of concentrations tested.

## Discussion

This study investigated the DNA damage induction by 4NQO at a low concentration range. 4NQO’s carcinogenic action is believed to be initiated by the enzymatic reduction of its nitro group (15,31). The reduction product 4HAQO of 4NQO reacts with DNA to form stable monoadducts with purine bases, which are considered to be responsible for its mutagenicity and genotoxicity (15).

The metabolically competent cell lines MCL-5 and AHH-1 were treated with 4NQO for 4h. 4NQO showed little to no significant increases in MN induction in AHH-1 and MCL-5 cells, even up to 55±5% toxicity. Further, in comparison to TK6 cells, AHH-1 and MCL-5 cells could be treated with a 10× higher concentration of 4NQO without reaching the 55±5% toxicity threshold suggesting their more robust nature. MCL-5 is a derivative of the AHH-1 cell line. Both cell lines are heterozygous for a p53 mutation at the interface between the codons 281 and 282 of exon 8 (21). The L5178Y cell line is known for its dysfunctional p53 activity, since they have a missense mutation in exon 5 on chromosome 11a (amino acid changes from a cysteine to an arginine) and a nonsense mutation in exon 4 on chromosome 11b (changing a glutamine to a stop codon) (22,23). L5178Y cells showed highly significant increases in MN induction and no decreases in cell viability could be observed at all (21,32). The tumour suppressor gene TP53 plays an important role in cellular integrity, with an integral function in transducing signals from damaged DNA to genes that control the cell cycle and lead to apoptosis. Consequently, the effects of DNA damaging agents could become significant at low concentrations, where p53 competent cell lines would induce DNA damage–dependent cell death or cell cycle blockage (32).

In addition to the p53 status of a cell, the participation of DNA repair pathways and differences in DNA repair capacity may influence the genotoxic responses in different cell lines. To our knowledge, there are no published data on variations in the repair abilities of the cell lines used in this study. However, previously conducted studies in our research facility showed minimal differences between mRNA levels of several alkylating agent relevant base excision repair enzymes in MCL-5, AHH-1 and TK6 cells (data not shown). Further experiments into the endogenous DNA repair protein levels and activity in the different cell lines need to be conducted to investigate the influence of variations in DNA repair on genotoxicity studies between cell lines. In addition, the differences in metabolic proficiencies of the various cell lines did not seem to have an impact on the observed dose responses, as 4NQO showed no increased genotoxicity in the cells with known metabolic competence. The chromosomal damage studies in p53 competent TK6 cells revealed that the short-term treatments (4h) without prolonged recovery time and extended treatments (24 and 48h) showed little to no chromosomal damage induction up to 55±5% cytotoxicity. However, extending the recovery time up to two cell cycles in the mononucleated assay increased the magnitude of MN induction at the higher concentration range. A study from Sobol *et al.* (30) showed that an extended recovery time after treatment with compounds with direct DNA reactivity in TK6 cells increased the magnitude of MN induction. Further it was proposed by Islaih *et al.* (33) that TK6 cells have prolonged cell cycle delay in response to genotoxins. Therefore, it might be possible that damaged cells are stalled rather than eliminated through apoptosis (30). Consequently, the extended recovery period would allow the stalled cells to progress into mitosis and hence to fix damage as a MN. In support of this, it was further noted in the present study that toxicity appeared to be higher in cells with the shorter recovery time than in cells with the extended recovery time. BMD modelling with covariate analysis provided further evidence for clear PoDs in most of the cell lines tested (no dose–response curve was observed in MCL-5 cells). The model estimated concentrations that produced predetermined biologically relevant increases in response over the control. The lower limit on the BMD_10_ is termed BMDL_10_ and is often considered an adequate PoD (28). The BMD covariate analysis (29) also allowed for ranking of 4NQO treatments in different cell lines to derive information on their genotoxic potency and showed that treatment of 4NQO in AHH-1 cells was the least potent, whereas the treatment in L5178Y was the most potent. Non-linear dose–response relationships for genotoxins have substantial implications for the setting of safe exposure levels and the understanding of cancer risk (34,35).

In conclusion, TK6 cells showed a smaller magnitude of MN induction and more cytotoxicity than the p53 mutated human lymphoblastoid cell lines AHH-1 and MCL-5 and in particular the mouse lymphoma cell line L5178Y, after 4NQO treatment. The sensitivity to MN induction and cytotoxicity is dependent on the cell type. However, cell lines with a p53 mutation are more likely to survive and replicate with DNA damage, which can lead to higher MN frequencies, since p53 plays an important role in cell responses, such as cell cycle arrest at the G1/S or G2/M phases, induced apoptosis and enhanced DNA repair in response to DNA damage (36). Cytoprotective mechanisms and DNA repair capacity after treatment with low concentrations with the genotoxin seem to play an important role in the observed dose responses.

In this study, it was shown that 4NQO was a weak inducer of chromosomal damage in human lymphoblastoid cells. Therefore, further investigations into the induction of gene mutations by 4NQO were undertaken. Increases in point mutations over the solvent control were observed in the HPRT assay, even at concentrations lower than those showing MN induction. Mutagenesis caused by 4NQO has been shown to be specific for base-paired substitutions, predominantly as G to A transitions, but also G to T conversions and rare substitutions of adenines (11). 4NQO induces three main adducts: dGuo-N2-AQO, dGuo-C8-AQO and dAdo-N6-AQO, with relative proportions in double-stranded DNA to be ~50, 30 and 10%, respectively (37). Further, it was shown that 4NQO induces apurinic/apyrimdinic sites and single-strand breaks (37). From this, it follows that further sequence analysis of 4NQO-induced mutations at the *hprt* locus in TK6 cells needs to be performed to determine the mutation spectra induced. However, it is most likely that the mutations induced by 4NQO are base substitutions.

The comet assay was used for detecting low levels of primary DNA damage. Increases in DNA damage induction in the comet assay were observed. The assay can detect both single-strand breaks as initial damage as well those developed from alkali-labile sites under alkaline conditions (38). As mentioned above, 4NQO causes apurinic/apyrimdinic sites and single-strand breaks (37). In previous studies, high levels of 8-OH-dG were found in 4NQO-treated cells, which implicated the involvement of ROS in the mutagenicity of 4NQO (11,15,31). Therefore, the lysed cells in the comet assay were incubated with the lesion-specific endonuclease hOGG1 to detect any oxidative DNA damage. Initial look at hOGG1 revealed little differences and the concentration levels investigated were in the cytotoxic range. Consequently, further experiments have to be undertaken to investigate the role of ROS in the mutagenicity of 4NQO.

In conclusion, this study was designed to assess the shape of the dose–response curve at low concentrations of 4NQO in human cells *in vitro* and to investigate the effect of different cell lines and study design on genotoxic potency and PoD metrics. The HPRT assay showed a PoD for DNA damage at a lower concentration than that of the MN assay and the comet assay, while the MN assay provided lower PoD metrics than the comet assay. From this it follows that it is most likely that 4NQO predominantly induces gene mutations, more so than DNA strand breaks or chromosomal damage. The difference between effects in the comet and MN assays could be due to variations in the type of DNA alterations that the test detects, while the comet assay detects primarily DNA lesions that are repairable, the MN assay detects irreparable lesions (39). From this it follows that the suitability of 4NQO as a positive control for genotoxicity testing has to be evaluated for every individual assay.

## Supplementary data

Supplementary Figure 1 is available at *Mutagenesis* Online.

## Funding

This study was supported by a Collaborative Award in Science and Engineering (CASE) studentship from Medical Research Council (MRC, grant number: G0900840-1/1) and AstraZeneca.

## Supplementary Material

Supplementary Data

## References

[1] Jenkins G. J. Zaïr Z. Johnson G. E. and Doak S. H (2010) Genotoxic thresholds, DNA repair, and susceptibility in human populations. Toxicology, 278, 305–310. 19932733 10.1016/j.tox.2009.11.016

[2] Doak S. H. Jenkins G. J. S. Johnson G. E. Quick E. Parry E. M. and Parry J. M (2007) Mechanistic influences for mutation induction curves following exposure to DNA-reactive carcinogens. Cancer Res., 67, 3904–3911. 17440105 10.1158/0008-5472.CAN-06-4061

[3] Thomas A. D. Jenkins G. J. S. Kaina B. Bodger O. G. Tomaszowski K.-H. Lewis P. D. Doak S. H. and Johnson G. E (2013) Influence of DNA repair on nonlinear dose-responses for mutation. Toxicol. Sci., 132, 87–95. 23288051 10.1093/toxsci/kfs341 PMC3576011

[4] Kirkland D. Pfuhler S. Tweats D. (2007) How to reduce false positive results when undertaking in vitro genotoxicity testing and thus avoid unnecessary follow-up animal tests: report of an ECVAM Workshop. Mutat. Res., 628, 31–55. 17293159 10.1016/j.mrgentox.2006.11.008

[5] Elhajouji A. Lukamowicz M. Cammerer Z. and Kirsch-Volders M (2011) Potential thresholds for genotoxic effects by micronucleus scoring. Mutagenesis, 26, 199–204. 21164203 10.1093/mutage/geq089

[6] Parry J. M. and Parry E. M (2012) Genetic Toxicology: Principles and Methods, Methods in Molecular Biology. Vol. 817 Springer, New York. 22250336

[7] Organisation of Economic Cooperation and Development (OECD). (2010). OECD Guideline for the Testing of Chemicals: In vitro Mammalian Cell Micronucleus Test (MNvit) 487.

[8] Nakahara W. Fukuoka F. and Sugimura T (1957) Carcinogenic action of 4-nitroquinoline-N-oxide. Gan, 48, 129–137. 13474141

[9] Endo H. Ono T. and Sugimura T (1971) Chemistry and Biological Actions of 4-Nitroquinoline 1-Oxide. Springer, Berlin–Heidelberg–New York.

[10] Sugimura T. Okabe K. and Nagao M (1966) The metabolism of 4-nitroquinoline-1-oxide, a carcinogen III. An enzyme catalyzing the conversion of 4-nitroquinoline-1-oxide to 4-hydroxyaminoquinoline-1-oxide in rat liver and hepatomas. Cancer Res., 26, 1717–1721. 4288552

[11] Bailleul B. Daubersies P. Galiegue-Zouitina S. and Loucheux-Lefebvre M.-H (1989) Molecular basis of 4-nitroquinoline 1-oxide carcinogenesis. Jpn. J. Cancer Res., 80, 691–697. 2511172 10.1111/j.1349-7006.1989.tb01698.x PMC5917829

[12] Tada M. and Tada M (1975) Seryl-tRNA synthetase and activation of the carcinogen 4-nitroquinoline 1-oxide. Nature, 255, 510–512. 166317 10.1038/255510a0

[13] Kohda K. Kawazoe Y. Minoura Y. and Tada M (1991) Separation and identification of N4-(guanosin-7-yl)-4-aminoquinoline 1-oxide, a novel nucleic acid adduct of carcinogen 4-nitroquinoline 1-oxide. Carcinogenesis, 12, 1523–1525. 1907226 10.1093/carcin/12.8.1523

[14] Jones C. J. Edwards S. M. and Waters R (1989) The repair of identified large DNA adducts induced by 4-nitroquinoline-1-oxide in normal or xeroderma pigmentosum group A human fibroblasts, and the role of DNA polymerases α or δ. Carcinogenesis, 10, 1197–1201. 2500268 10.1093/carcin/10.7.1197

[15] Arima Y. Nishigori C. Takeuchi T. Oka S. Morimoto K. Utani A. and Miyachi Y (2006) 4-Nitroquinoline 1-oxide forms 8-hydroxydeoxyguanosine in human fibroblasts through reactive oxygen species. Toxicol. Sci., 91, 382–392. 16547075 10.1093/toxsci/kfj161

[16] Nunoshiba T. and Demple B (1993) Potent intracellular oxidative stress exerted by the carcinogen 4-nitroquinoline-N-oxide. Cancer Res., 53, 3250–3252. 8391920

[17] Kanojia D. and Vaidya M. M (2006) 4-Nitroquinoline-1-oxide induced experimental oral carcinogenesis. Oral Oncol., 42, 655–667. 16448841 10.1016/j.oraloncology.2005.10.013

[18] Crofton-Sleigh C. Doherty A. Ellard S. Parry E. M. and Venitt S (1993) Micronucleus assays using cytochalasin-blocked MCL-5 cells, a proprietary human cell line expressing five human cytochromes P-450 and microsomal epoxide hydrolase. Mutagenesis, 8, 363–372. 8377657 10.1093/mutage/8.4.363

[19] Morris S. M. Manjanatha M. G. Shelton S. D. Domon O. E. McGarrity L. J. and Casciano D. A (1996) A mutation in the p53 tumor suppressor gene of AHH-1 tk+/− human lymphoblastoid cells. Mutat. Res., 356, 129–134. 8841477 10.1016/0027-5107(96)00133-9

[20] Dobo K. L. Eastmond D. A. and Grosovsky A. J (1997) The influence of cellular apoptotic capacity on N-nitrosodimethylamine-induced loss of heterozygosity mutations in human cells. Carcinogenesis, 18, 1701–1707. 9328164 10.1093/carcin/18.9.1701

[21] Guest R. D. and Parry J. M (1999) P53 integrity in the genetically engineered mammalian cell lines AHH-1 and MCL-5. Mutat. Res., 423, 39–46. 10029673 10.1016/s0027-5107(98)00223-1

[22] Storer R. D. Kraynak A. R. McKelvey T. W. Elia M. C. Goodrow T. L. and DeLuca J. G (1997) The mouse lymphoma L5178Y Tk+/− cell line is heterozygous for a codon 170 mutation in the p53 tumor suppressor gene. Mutat. Res., 373, 157–165. 9042396 10.1016/s0027-5107(96)00227-8

[23] Clark L. S. Harrington-Brock K. Wang J. Sargent L. Lowry D. Reynolds S. H. and Moore M. M (2004) Loss of P53 heterozygosity is not responsible for the small colony thymidine kinase mutant phenotype in L5178Y mouse lymphoma cells. Mutagenesis, 19, 263–268. 15215324 10.1093/mutage/geh024

[24] Crespi C. L. Gonzalez F. J. Steimel D. T. Turner T. R. Gelboin H. V. Penman B. W. and Langenbach R (1991) A metabolically competent human cell line expressing five cDNAs encoding procarcinogen-activating enzymes: application to mutagenicity testing. Chem. Res. Toxicol., 4, 566–572. 1793807 10.1021/tx00023a013

[25] Brüsehafer K. Rees B. J. Manshian B. B. Doherty A. T. O’Donovan M. R. Doak S. H. and Jenkins G. J. S (2014) Chromosome breakage induced by the genotoxic agents mitomycin C and cytosine arabinoside is concentration and p53 dependent. Toxicol. Sci., 140, 94–102. 24675086 10.1093/toxsci/kfu058

[26] Smith C. C. O’Donovan M. R. and Martin E. A (2006) hOGG1 recognizes oxidative damage using the comet assay with greater specificity than FPG or ENDOIII. Mutagenesis, 21, 185–190. 16597659 10.1093/mutage/gel019

[27] Johnson G. E. Soeteman-Hernandez L. G. Gollapudi B. B. (2014) Derivation of point of departure (PoD) estimates in genetic toxicology studies and their potential applications in risk assessment. Environ. Mol. Mutagen., 55, 609–623. 24801602 10.1002/em.21870 PMC6710644

[28] Gollapudi B. B. Johnson G. E. Hernandez L. G. (2013) Quantitative approaches for assessing dose-response relationships in genetic toxicology studies. Environ. Mol. Mutagen., 54, 8–18. 22987251 10.1002/em.21727

[29] Slob W. and Setzer R. W (2013) Shape and steepness of toxicological dose–response relationships of continuous endpoints. Crit. Rev. Toxicol., 44, 270–297. 24252121 10.3109/10408444.2013.853726

[30] Sobol Z. Homiski M. L. Dickinson D. A. (2012) Development and validation of an in vitro micronucleus assay platform in TK6 cells. Mutat. Res., 746, 29–34. 22445949 10.1016/j.mrgentox.2012.02.005

[31] Stankowski L. F. Jr Roberts D. J. Chen H. Lawlor T. McKeon M. Murli H. Thakur A. and Xu Y (2011) Integration of a Pig-a, micronucleus, chromosome aberration, and comet assay endpoints in a 28-day rodent toxicity study with 4-nitroquinoline-1-oxide. Environ. Mol. Mutagen., 52, 738–747. 22020836 10.1002/em.20692

[32] Pucci B. Kasten M. and Giordano A (2000) Cell cycle and apoptosis. Neoplasia, 2, 291–299. 11005563 10.1038/sj.neo.7900101 PMC1550296

[33] Islaih M. Halstead I. A. Kadura B. (2005) Relationships between genomic, cell cycle, and mutagenic responses of TK6 cells exposed to DNA damaging chemicals. Mutat. Res., 578, 100–116. 16109433 10.1016/j.mrfmmm.2005.04.012

[34] Jenkins G. J. Doak S. H. Johnson G. E. Quick E. Waters E. M. and Parry J. M (2005) Do dose response thresholds exist for genotoxic alkylating agents? Mutagenesis, 20, 389–398. 16135536 10.1093/mutage/gei054

[35] Seager A. L. Shah U.-K. Mikhail J. M. (2012) Pro-oxidant induced DNA damage in human lymphoblastoid cells: homeostatic mechanisms of genotoxic tolerance. Toxicol. Sci., 128, 387–397. 22539617 10.1093/toxsci/kfs152 PMC3493188

[36] Hashimoto K. Nakajima Y. Uematsu R. Matsumura S. and Chatani F (2011) Involvement of p53 function in different magnitude of genotoxic and cytotoxic responses in in vitro micronucleus assays. Mutat. Res., 726, 21–28. 21855649 10.1016/j.mrgentox.2011.07.009

[37] Inga A. Iannone R. Degan P. Campomenosi P. Fronza G. Abbondandolo A. and Menichini P (1994) Analysis of 4-nitroquinoline-1-oxide induced mutations at the hprt locus in mammalian cells: possible involvement of preferential DNA repair. Mutagenesis, 9, 67–72. 8208132 10.1093/mutage/9.1.67

[38] Kawaguchi S. Nakamura T. Yamamoto A. Honda G. and Sasaki Y. F (2010) Is the comet assay a sensitive procedure for detecting genotoxicity? J. Nucleic Acids, 2010, 1–8. 10.4061/2010/541050 PMC2967831 21052491

[39] Valentin-Severin I. Le Hegarat L. Lhuguenot J. C. Le Bon A. M. and Chagnon M. C (2003) Use of HepG2 cell line for direct or indirect mutagens screening: comparative investigation between comet and micronucleus assays. Mutat. Res., 536, 79–90. 12694748 10.1016/s1383-5718(03)00031-7

