# Supplementary material for: The clastogenicity of 4NQO is cell-type dependent and linked to cytotoxicity, length of exposure and p53 proficiency
Source: Mutagenesis. 2015 Sep 11;31(2):171–80. doi: 10.1093/mutage/gev069 (PMC4748179; doi:10.1093/mutage/gev069)
Supplement: Supplementary Data [file supp_31_2_171__index.html]

The clastogenicity of 4NQO is cell-type dependent and linked to cytotoxicity, length of exposure and p53 proficiency — Supplementary Data 

# The clastogenicity of 4NQO is cell-type dependent and linked to cytotoxicity, length of exposure and p53 proficiency

## Supplementary Data

Data files

- Supplementary Data - Supplementary Data
